# Validation and assessment of an antibiotic-based, aseptic decontamination manufacturing protocol for therapeutic, vacuum-dried human amniotic membrane

**DOI:** 10.1038/s41598-019-49314-7

**Published:** 2019-09-06

**Authors:** Nagi M. Marsit, Laura E. Sidney, Emily R. Britchford, Owen D. McIntosh, Claire L. Allen, Waheed Ashraf, Roger Bayston, Andrew Hopkinson

**Affiliations:** 1 Academic Ophthalmology, Division of Clinical Neuroscience, School of Medicine, University of Nottingham, Nottingham, UK; 2 Biomaterials Related Infection Group, Academic Orthopaedics, School of Medicine, University of Nottingham, Nottingham, UK

**Keywords:** Antimicrobials, Corneal diseases, Translational research

## Abstract

Amniotic membrane (AM) is used to treat a range of ophthalmic indications but must be presented in a non-contaminated state. AM from elective caesarean sections contains natural microbial contamination, requiring removal during processing protocols. The aim of this study was to assess the ability of antibiotic decontamination of AM, during processing by innovative low-temperature vacuum-drying. Bioburden of caesarean section AM was assessed, and found to be present in low levels. Subsequently, the process for producing vacuum-dried AM (VDAM) was assessed for decontamination ability, by artificially loading with *Staphylococcus epidermidis* at different stages of processing. The protocol was highly efficient at removing bioburden introduced at any stage of processing, with antibiotic treatment and drying the most efficacious steps. The antibacterial activity of non-antibiotic treated AM compared to VDAM was evaluated using minimum inhibitory/biocidal concentrations (MIC/MBC), and disc diffusion assays against Meticillin-resistant *Staphylococcus aureus*, Meticillin-resistant *S*. *epidermidis*, *Escherichia coli*, *Pseudomonas aeruginosa* and *Enterococcus faecalis*. Antibacterial activity without antibiotic was low, confirmed by high MIC/MBC, and a no inhibition on agar lawns. However, VDAM with antibiotic demonstrated effective antibacterial capacity against all bacteria. Therefore, antibiotic decontamination is a reliable method for sterilisation of AM and the resultant antibiotic reservoir is effective against gram-positive and –negative bacteria.

## Introduction

Treatment of ocular surface disease, ulcers and infections remains a challenge. Severe bacterial ocular infections are commonly treated with corticosteroids and an intensive course of antibiotics^1,2^. However, the toxic side-effects of antibiotics can be harmful to epithelial cells of the cornea^2,3^.

Human amniotic membrane (AM), processed and preserved for therapeutic application is reported to possess anti-inflammatory, anti-scarring, anti-angiogenic and anti-microbial properties, which are valued for their role in wound healing and prevention of post-surgery complications^4–7^. Application of AM in the treatment of corneal disease has been reported to reduce infection-causing microorganisms and accelerate healing of infected corneas^8^, having been shown to be superior to antibiotic treatment alone^7,9^.

AM intervention is an option that mitigates the prolonged use of topical, broad-spectrum antibiotics, preventing steroid-related complications. Studies supporting the use of AM for the treatment of corneal ulcers show significantly enhanced epithelial healing and a reduction in inflammation, corneal scarring, neovascularization, and infection complications^5,7,9^. Although the mechanism of action of AM is not well explained, it is often attributed to bioactive substances such as growth factors, pro-inflammatory cytokines and anti-inflammatory cytokines, that enhance the function of structural constituents, working as a substrate for local cell migration and adhesion^10^.

Freshly collected AM is rarely used for clinical treatment due to post-transplant complications^11^, increased probability of disease transmission, and short shelf-life. Therefore, most clinically used AM is subject to processing and preservation steps prior to use^10^. Cryopreservation^12^, heat-drying^13^ and freeze-drying^14^ are the most common methods for AM preservation. Although associated with low post-transplantation infection complications^15^, cryopreservation has been shown to damage the amnion^16^ which impairs the viability and proliferative capacity of epithelial cells^17^, decrease levels of many bioactive substances including epidermal growth factors (EGF) and transforming growth factors (TGF), and is constrained by distribution difficulties^18^. Dried AM followed by radiation sterilisation has been shown to be degraded with reduced growth factor content^14^. This effect on the stability and function of biological molecules such as growth factors, cytokines and structural proteins, affects the quality of the AM as a biological treatment for eye injury, and justifies the development of an appropriate approach for AM preservation.

Due to potential degradation by terminal sterilisation techniques^19^, AM is often aseptically prepared in order to ensure no contamination is introduced during processing and to limit the spread of any natural bioburden. *In vivo* during pregnancy, AM is considered sterile, however, potential microbial contamination originates from skin and vaginal flora during delivery of the placenta during birth, with the retrieving staff being an additional source of contamination^20^. Therefore, any manufacturing process must involve steps to effectively decontaminate such bioburden and allow for long-term preservation of AM.

Fresh AM processing generally involves a series of washing steps in physiological solutions to remove raw contamination and blood. Subsequent preservation via heat, freeze-drying, or deep freezing at below −60 °C are applied to suppress water activity to maintain tissue stability, and prevent microbial growth. However, to address bioburden contamination, antibiotics are typically added to the collection medium or storage medium for frozen AM^20,21^. For dried AM products, vacuum-packing followed by radiation sterilisation is often applied to ensure complete microbial inactivity^22^.

The sterility and integrity of final manufactured AM is necessary for safe and direct application. However, maintaining AM sterility, quality and functional properties during processing or storage is a challenge. Manufacturing AM in the presence of selected antibiotic/antifungal agents can have several advantageous features, including avoiding deleterious effects on the matrix and biomolecules caused by terminal sterilisation, providing sterility, and inadvertently working as a potential antibiotic reservoir that offers a prophylactic treatment following transplantation.

The aim of this study was to validate the microbial decontamination potential of a novel vacuum-drying, aseptic manufacturing technique, and to then investigate further the lasting anti-microbial effects of antibiotics added during processing over long-term storage. We identify the level of microbiological contamination, or bioburden, of elective caesarean section (ECS) AM, and then go on to assess the robustness of decontamination during our optimised, innovative protocol for preserving AM, using vacuum-drying and a broad spectrum antibiotic/antifungal cocktail^18^. This is done by artificially loading AM with high levels of *Staphylococcus epidermidis*, a skin floral species likely to contaminate amnion^23^, and investigating the decontamination efficacy of the washing, antibiotic treatment and vacuum-drying steps by counting the surviving colony forming units (CFU) using a spread plate method. The implications of biological and physical antimicrobial activity of fresh AM and the inadvertent antibiotic reservoir effect of vacuum-dried AM (VDAM) against strains of gram-positive and gram-negative bacteria are then studied using minimum inhibitory and biocidal concentrations (MIC/MBC) and disc-diffusion assays.

## Results

### Natural bioburden of fresh ECS AM

Results demonstrating the normal (unloaded) floral contamination of fresh ECS AM can be seen in Table 1. Results showed that 40% of the fresh AM obtained from caesarean section placentae were not contaminated with detectable bioburden. Of the remaining 60%, any contamination was predominantly identified as *Neisseria cinerea* or *Micrococcus* spp. Enhanced anaerobic incubation for seven days showed increased growth of *Cutibacterium acnes* bacteria in samples that were negative at aerobic conditions.

**Table 1:** Bioburden assessment and identification of natural bioburden of 10 freshly collected ECS AM (NG – no growth).

AM	Bacterial Identification			
	Blood Agar Culture Plates		Basal Broth Culture	
	Aerobic (48 h)	Anaerobic (7 days)	Aerobic(48 h)	Anaerobic (7 days)
1	*Neisseria cinerea*	NG	NG	NG
2	*Micrococcus* spp.	*Cutibacterium acnes*	*Micrococcus* spp.	*P*. *acnes*
3	NG	30 CFU/mL	NG	*Cutibacterium acnes P*. *acnes Staph*. *schleiferi*
4	NG	NG	NG	NG
5	NG	NG	*Micrococcus* spp.	NG
6	NG	NG	NG	NG
7	NG	NG	*Micrococcus* spp.	NG
8	NG	NG	NG	*Micrococcus* spp.
9	NG	NG	NG	NG
10	NG	NG	NG	NG

### Validation of vacuum-drying protocol with artificially loaded bioburden

**Figure 1:**
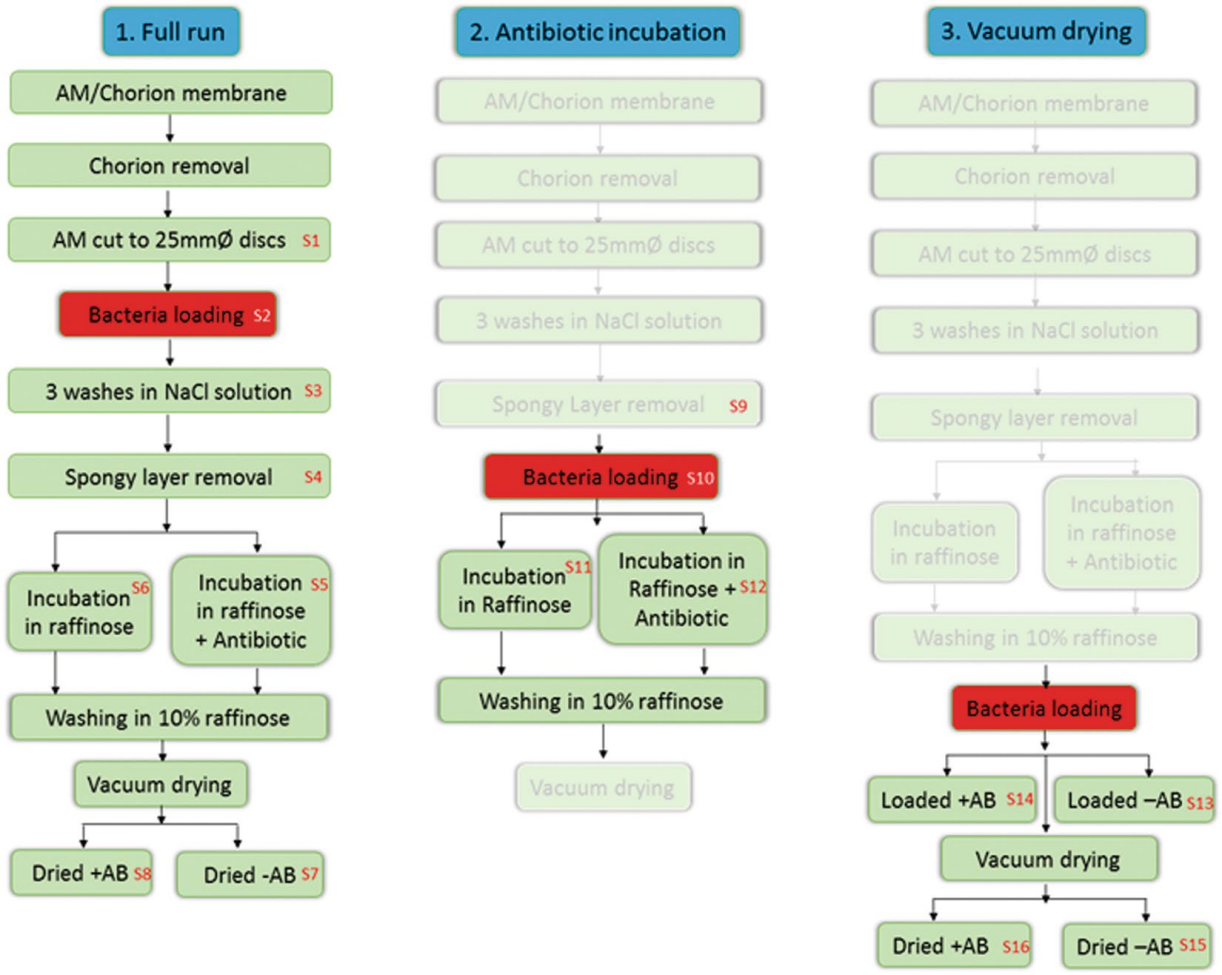
Schematic diagram showing sampling procedures for the *S*. *epidermidis* loaded bioburden experiment. Discs of 25 mm diameter were collected at each point numbered in red (S1–S15). Experiment was performed on 5 separate donors.

Due to low and inconsistent levels of natural bioburden on ECS AM, we artificially loaded 5 donated amnion with high bioburden (10^6^ CFU/mL), at different stages of processing, to effectively test the decontamination efficacy of the VDAM process. Figure 1, shows a schematic of the bacterial loading and sampling procedure performed. Unloaded samples of each donor (n = 3 per donor) were tested before washing in NaCl and no detectable contamination was seen.

### Decontamination efficacy of full manufacturing run

**Figure 2:**
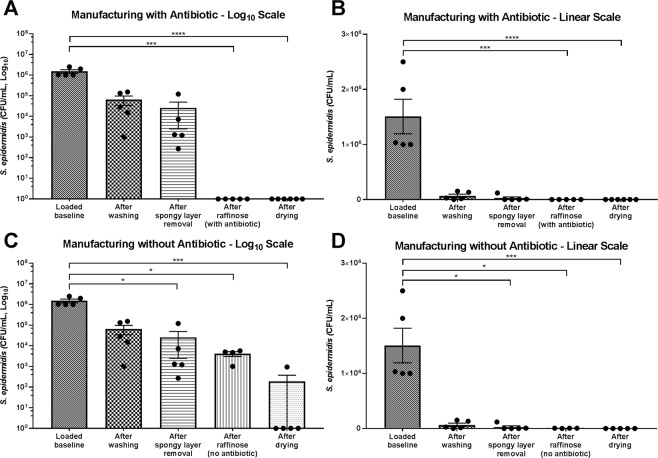
Effect of VDAM processing on reduction of artificially loaded *S*. *epidermidis* bacterial counts. Loading of 100 μL of 10^6^ CFU/mL *S*. *epidermidis* was performed before washing procedures in 0.9% NaCl. AM samples were taken throughout processing and assessed for CFU/mL. Manufacturing was performed with antibiotic included in the raffinose step (**A**) graph shown with log_10_ scale, (**B**) graph shown with linear scale. Manufacturing with antibiotic included (**C**) graph shown with log_10_ scale, (**D**) graph shown with linear scale. Data shows individual experiments performed with 5 different donors and is represented as mean ± SEM. Data compared by one-way ANOVA, Kruskal-Wallis test followed by multiple comparison uncorrected Dunn’s test. Statistical significance vs. baseline represented by *p ≤ 0.05, ***p ≤ 0.001, ****p ≤ 0.0001.

The efficacy of the entire processing procedure to detach and devitalise bacterial contamination loaded at the start was examined by counting CFU/mL after each processing step (Fig. 2, graphs shown on log_10_ and linear scales). AM samples loaded with *S*. *epidermidis* showed significant successive reduction from the original load of 10^6^ CFU/mL as processing progressed. Washing three times in NaCl reduced the bacterial load by almost one log cycle (post wash, 95.65%) and the removal of the spongy layer contributed to further lowering the remaining microbial load by 60.53%. Incubating the AM in raffinose solution containing a broad spectrum antibiotic cocktail (components listed in Table 2), caused the bioburden to be essentially eliminated, with no growth detected after raffinose/antibiotic incubation, or after the combined processing of raffinose/antibiotic incubation and vacuum drying (Fig. 2A,B). Incubating in raffinose without antibiotic (Fig. 2C,D) resulted in a further reduction of the remaining bacteria by 87.29% and continuing to the drying step without antibiotic treatment excluded more CFU by 94.26%.

**Table 2:** Concentrations and activity of the antibiotic/antifungal cocktail used in this study.

Antibiotic	Concentration	% w/v	Activity
Gentamicin	200 mg/mL	15–20	Gram-positive, gram-negative
Imipenem	10 mg/mL	5–10	Gram-positive, gram-negative
Polymyxin B	10 mg/mL	5–10	Gram-negative
Vancomycin	2.5 mg/mL	<0.5	Gram-positive
Nystatin	125,000 U/mL	<0.05	Antifungal

### Efficacy of raffinose/antibiotic incubation step

**Figure 3:**
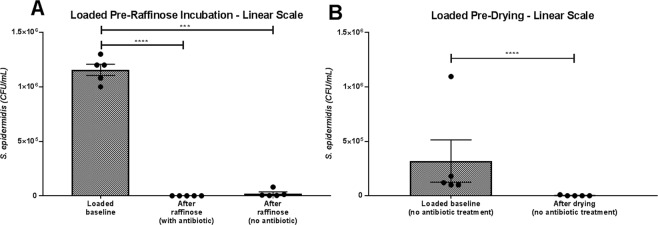
Efficiency of antibiotic incubation and drying steps in reducing artificially loaded *S*. *epidermidis* bacterial counts. (**A**) Loading of 100 μL of 10^6^ *S*. *epidermidis* was performed before raffinose incubation with/without antibiotic. AM samples were taken after loading and after incubation and assessed for CFU/mL. (**B**) Loading of 100 μL of 10^6^ CFU/mL *S*. *epidermidis* was performed after raffinose incubation with/without antibiotic and before drying. AM samples were taken after loading and after drying and assessed for CFU/mL. Only samples processed without antibiotic are shown, as no colonies were seen either in the loaded baseline or after drying. Data represents experiments performed on 5 different donors and is represented as mean ± SEM. Data compared by one-way ANOVA, Kruskal-Wallis test followed by multiple comparison uncorrected Dunn’s test. Statistical significance vs. baseline represented by ***p ≤ 0.001, ****p ≤ 0.0001.

To test the efficacy of the raffinose incubation step with and without antibiotics, loading the AM was performed after the spongy layer removal step (Fig. 3A). This removes any effect that previous manufacturing steps had on the bacterial reduction. Incubation in raffinose containing antibiotic effectively eliminated the loaded bacteria. Raffinose incubation without antibiotic removed the majority of loaded bacteria resulting in a 98.17% reduction, similar to that seen during the full run experiment.

### Efficacy of vacuum-drying step

To test the efficacy of the vacuum-drying step, samples were loaded with *S*. *epidermidis* after the raffinose incubation and was performed with and without antibiotic (Fig. 3B). When antibiotic had been included in the process prior to loading, no CFU were found either in the loaded baseline or after drying (not shown on graph). When processed without antibiotics, the loaded baseline was lower than seen in previous experiments, indicating an inefficiency of loading at this point of processing. Subsequent drying then resulted in a 99.41% reduction of loaded bioburden.

### Antibacterial efficacy of AM

#### MIC/MBC test

MIC tests showed that washed AM extract without antibiotic treatment did not exert any consistent antimicrobial activity. Bacterial growth was observed across the 96-well plates (Table 3); therefore, the MIC could not be determined. When the AM was incubated with antibiotic, the MIC results for each donor ranged from 0.78 µg/mL to 12.5 µg/mL, and the MBC results ranged from 6.25 µg/mL to 25 µg/mL. These results indicate that the AM itself has very low natural antimicrobial properties but inclusion of the antibiotics in the process, even in small concentrations, can cause bacterial elimination.

**Table 3:** MIC (Minimum Inhibitory Concentration) and MBC (Minimum Bactericidal Concentration) of serially diluted AM extracts treated with raffinose, with and without antibiotic treatment. Results are shown as average values from three separate experiments.

Processed without antibiotics						
Bacterial Strain	Sample 1		Sample 2		Sample 3	
	MIC (µg/mL)	MBC (µg/mL)	MIC (µg/mL)	MBC (µg/mL)	MIC (µg/mL)	MBC (µg/mL)
*S*. *aureus*	100	100	100	100	25	25
*S*. *epidermidis*	50	100	100	100	100	100
*C*. *diphtheria*	100	100	100	100	100	100
*Moraxella*	100	100	100	100	50	100
*S*. *aureus*	6.25	6.25	3.13	6.25	6.25	12.5
*S*. *epidermidis*	6.25	12.5	0.78	3.13	6.25	12.5
*C*. *diphtheria*	12.5	12.5	12.5	12.5	3.13	6.25
*Moraxella*	3.13	6.25	6.25	6.25	12.5	25

#### Disc diffusion assay

**Figure 4:**
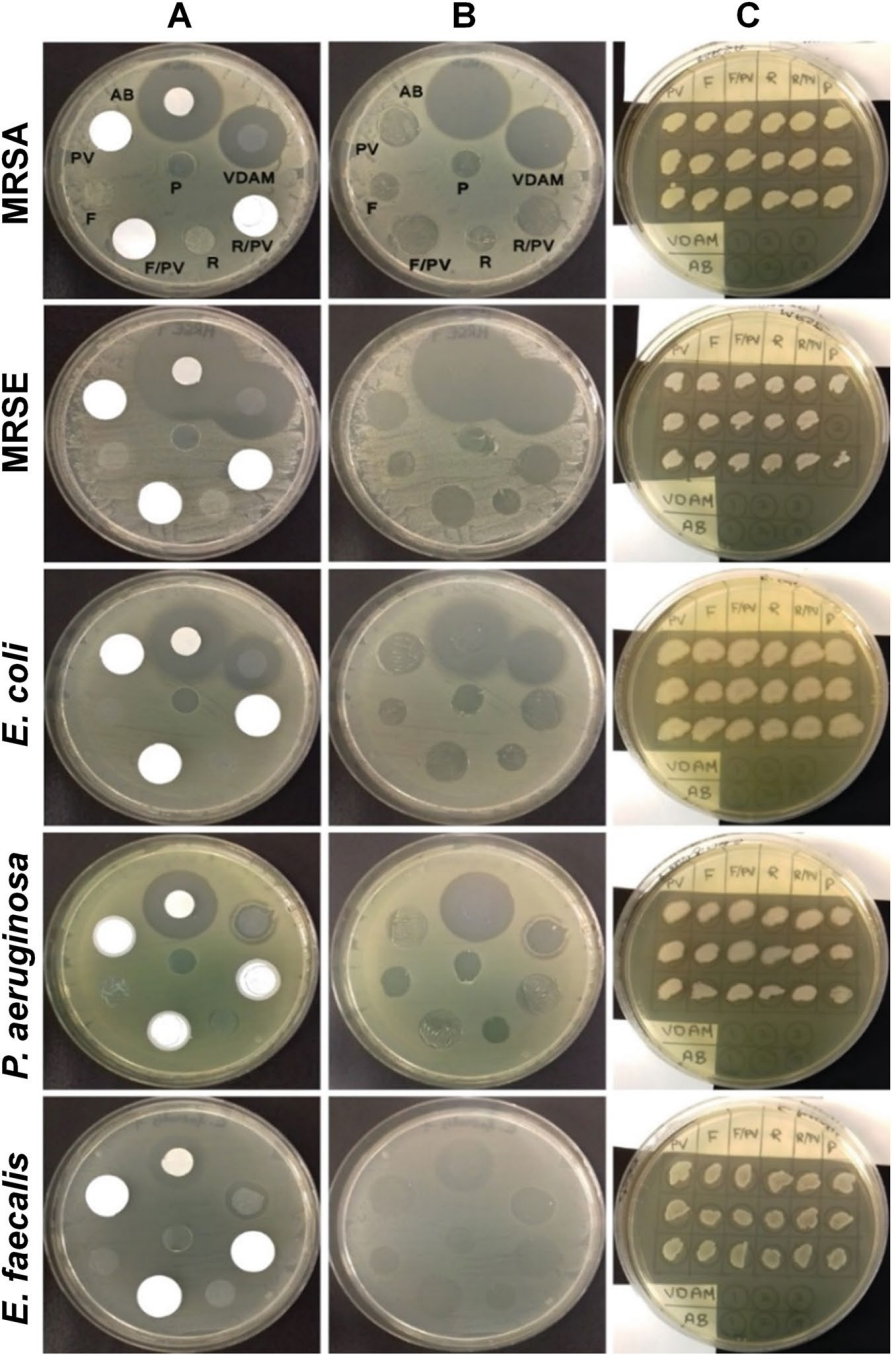
Antibacterial activity of various amniotic membrane preparations. Discs of 10 mm diameter were prepared of VDAM; non-raffinose and non-antibiotic treated AM (**F**), non-antibiotic treated AM (R) and antibiotic soaked Whatman no. 3 filter paper (AB) as a positive control. Polyvinylidene fluoride (PVDF) transfer membrane discs (PV) were used as a control to the following disc samples: F over PVDF (F/PV) and R over PVDF (R/PV). The transparent plastic material (P) was used as a carrier to transfer VDAM onto new for a further incubation period. All discs were laid on lawns of MRSA, MRSE, *E*. *coli*, *P*. *aeruginosa* and *E*. *faecalis*. Images show representative MHA plates after overnight incubation at 35 °C. (**A**) Bacterial lawns with discs still present. (**B**) Same plates after removal of samples. (**C**) Streaks from areas underneath discs, shown in B, onto lawns of corresponding bacteria on MHA plates after 35 °C overnight incubation. Experimental error caused MRSE sample P2 not to be streaked. Experiment conducted on three different donors.

Results from the disc diffusion assay can be seen in Fig. 4. The only samples to form a zone of inhibition (ZOI) against all 5 bacteria studied were VDAM and the positive control (AB). Effects from VDAM on MRSA (ZOI: 10.74 mm) and MRSE (ZOI: 15.87 mm) were higher than on those of *E*. *coli* (ZOI: 8.82 mm), *P*. *aeruginosa* (ZOI: 2.48 mm) and *E*. *faecalis* plates (ZOI: 1.96 mm). All samples of VDAM processed without antibiotic (R) VDAM, processed without raffinose or antibiotic (F), and the PVDF sample discs were not bactericidal to the five strains and did not produce ZOI. However, once the discs were removed there was reduced bacterial growth found below the sample (Fig. 4B). To test the number of bacteria that remained once the discs had been removed, swabs were taken and performed on a new plate (Fig. 4C), before visual assessment. VDAM and AB controls had eliminated all bacteria found directly below them. All other samples showed that viable bacteria remained below where the disc had been placed.

**Figure 5:**
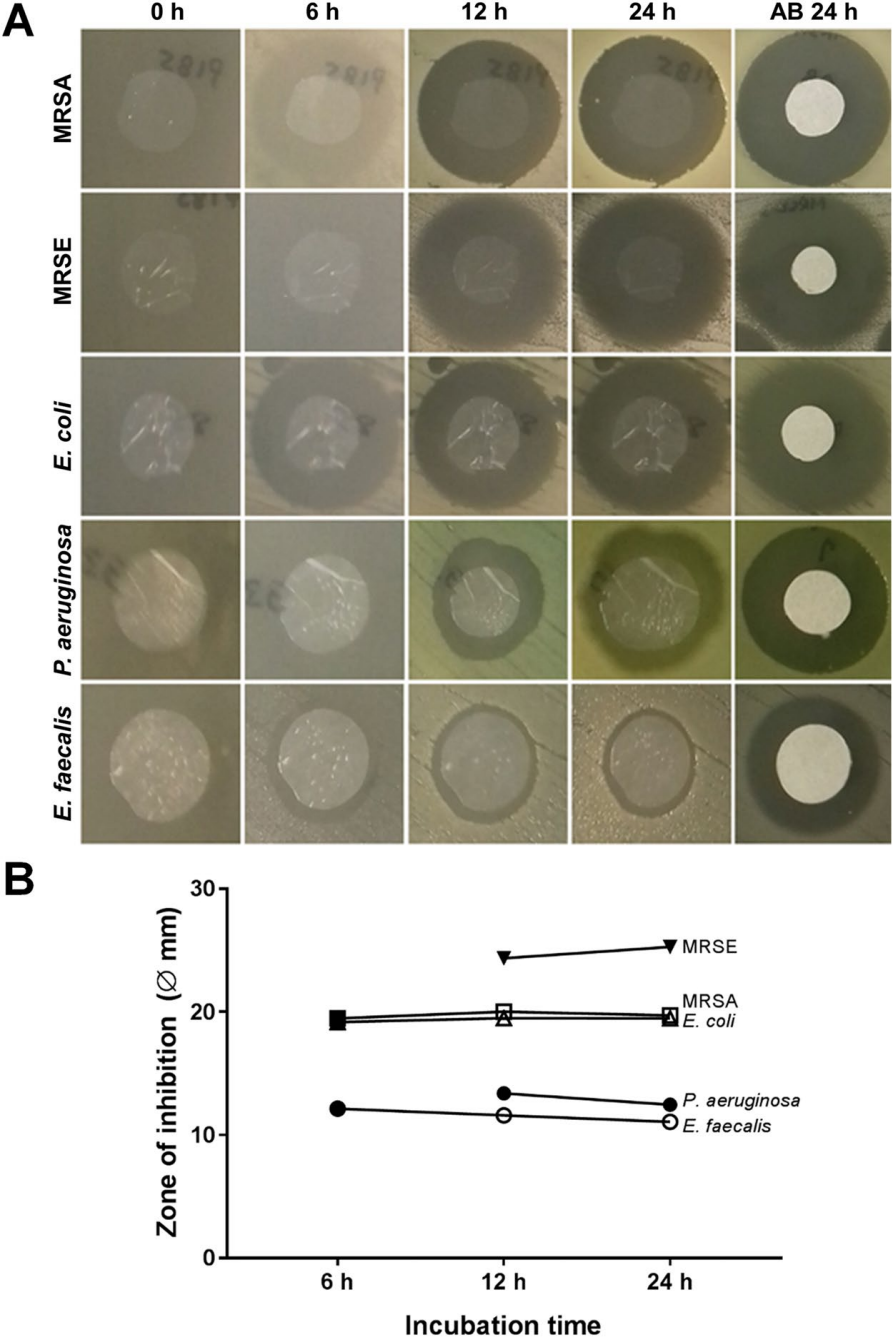
Antibiotic release from VDAM over time. (**A**) Antibiotic release from 10 mm diameter discs of VDAM placed on bacterial lawns. Representative images show zones of inhibition of the same disc after 6, 12 and 24 hours of incubation at 35 °C. Filter paper discs impregnated in antibiotic were used as control in every plate. Experiment performed on triplicate samples from 3 different donors and repeated 3 times for all bacteria. (**B**) Quantification of the zone of inhibition (including disc diameter) at the 6, 12 and 24 hour time points.

Diffusion assays performed over time (Fig. 5) showed that the antibacterial effects of the antibiotic release from VDAM could only be seen in full effect after certain time points. At 6 hours incubation, ZOI were measured from MRSA, *E*. *coli* and *E*. *faecalis*, but not from MRSE and *P*. *aeruginosa*. Notably, the antibiotic content of VDAM is released rapidly in the medium before the bacteria show a distinct growth in the first 6 hours.

#### Sterility test of VDAM held in long-term storage

VDAM samples that had been stored, vacuum-packed, at 4–6 °C for over two years were placed into TSB or thioglycolate broth medium to test for contaminant growth. Of the 21 samples of 25 mm diameter that were tested, only one sample reported any growth, and only of aerobic bacteria. Two others from the same donor did not show any contamination. Of the 30 samples of 10 mm diameter, 1 sample showed contaminant growth of both aerobic and anaerobic bacteria. Once again, two other samples from the same donor, processed identically, showed no growth. These samples were judged to have been contaminated during test handling.

## Discussion

The natural floral contamination of fresh AM obtained by ECS in the Queens’s Medical Centre, Nottingham University Hospitals NHS trust, was shown to be considerably lower than literature reported from normal vaginal deliveries^20,24,25^. 40% of the tested AM showed no signs of bioburden contamination, similar to results previously reported (39%)^20^. Of those that demonstrated contamination, only *Micrococcus* spp, *Propionibacterium acnes*, *S*. *schleiferi* and *N*. *cinerea* were identified, most likely from skin contact of the placenta during delivery. Previous reports have identified up to 12 species in their surveys including the ones named in our study^20,24,26^. *Staphylococcus* spp have been identified as the most prevalent contamination of AM^15,20,26^. This study indicates that the risk of major contamination from AM obtained through ECS is low, reducing the risk of contaminated AM being transplanted to the eye. Additional results from PBS samples collected immediately from raw AM before washing (incubated overnight at 35 °C on BA) contained no bioburden (n = 15 from 5 donors), demonstrating that fresh AM could be bacteriostatic before processing.

VDAM was developed to be stored dry at room temperature in a stable state until use, avoiding the cold chain storage and distribution challenges associated with fresh and frozen AM. A bioburden-free VDAM final product was confirmed during investigation into our protocol, and long-term sterility test results confirm sterility of end-product samples, even when artificially high levels of *S*. *epidermidis* were loaded during different stages of processing. *S*. *epidermidis* is an ocular pathogen and was selected as a model of *staphylococcus genera* which is one of the most prevalent causes of bacterial keratitis^27^. It is considered to be one of the skin floral species most likely to contaminate AM, and has also been shown to adhere well to fresh AM^23^.

Our results revealed that three successive washes in 0.9% NaCl reduced bioburden by 95.65%, in agreement with Germain *et al*., (2010) who reported that frequent washing can reduce bioburden in processed heart valves^28^. Most AM preparation protocols use repeated washing in physiological saline to remove blood and immunogens, as well as remains of amniotic fluid. However, we have found that to retain the integrity of the epithelial surface and matrix biomolecules appropriate washing times have to be optimised^18^. As an added safety precaution for preserved AM, different antibiotic/antimycotic combinations can be used to cover a wide range of gram-positive, gram-negative bacteria and fungi, including penicillin, streptomycin, neomycin, amphotericin B, gentamycin, imipenem, nystatin, polymyxin and vancomycin^29,30^. We used a combination of the latter five agents, a cocktail widely used in the UK for tissue allograft banking^31^. Reports show that antibiotic solutions used for tissue allograft preservation can be stored for up to one-year storage at −20 and −80 without effect on potency. Dried forms of these antibiotics can withstand storage at 37 °C for up to 12 months^32^.

The microbicide activity of antibiotic treatment was found to be more effective at 37 °C than at 4 °C or 22 °C^28^. Long antibiotic incubation, such as 12 hours at 37 °C, has been shown to be more efficient, but could potentially compromise the cell viability of tissue, including AM^28,33,34^. The current study showed that a two-hour incubation at 37 °C resulted in full eradication of bacteria loaded just before the antibiotic treatment step and that longer incubation times are not required. The antibiotic cocktail was capable of completely eradicating a high load of bacteria.

AM prepared in antibiotics can serve as a reservoir during transplantation, particularly effective against microbial keratitis frequently caused by different bacteria strains including the conjunctival normal flora *S*. *epidermidis*^27,35,36^. AM can absorb and release antibiotics after rehydration on a wet surface such as agar medium or the corneal surface. Our results showed that the pattern of VDAM release of antibiotic was consistent with the antibiotic control discs, with rapid release at the beginning of application (6 hours) without considerable change in ZOI after 12 and 24 hours of incubation on bacterial lawns. AM has been shown to have slow antibiotic release over seven hours, dependent on the time of AM soaking in antibiotic^37^, however the release profile may be different for different antibiotics^38^.

Dehydration of AM by air, heat or freeze-drying (lyophilisation) can be applied after washing procedures to decrease the water content of AM, hindering the spread of contamination during storage, and extending the shelf-life. Drying is applied as a final step before packaging, and has been found to maintain AM structural and biological molecules^39^. AM desiccated to lower or equal to 5% water content prevents microbial growth, thus there is less chance for product contamination. To confirm this, we showed that vacuum-drying alone, without antibiotic treatment, was efficient in removing 94.26% of bacteria, when samples were loaded after the raffinose washing step. Drying in our protocol is primarily applied to AM as a method of preservation and not as a decontamination step, however, so as it was not efficient in reducing all of the bacterial load it would only ever be used as an adjunct to antibiotic treatment for decontamination.

Previous studies have shown that AM antibacterial function after washing or prolonged rehydration was significantly reduced^40^. No ZOI was observed around washed AM against *S*. *epidermidis*^38^. Tehrani *et al*. (2013), showed a decrease in antibacterial activity against *S*. *aureus*, *P*. *aeruginosa* and *E*. *coli* of freeze dried AM after 2 hours rehydration, and after 3 times rinsing in PBS^41^. Our MIC/MBC *in vitro* study of AM antibacterial activity showed that AM processed without antibiotics has little effect on *S*. *aureus*, *S*. *epidermidis*, *C*. *diphtheria* and *Moraxella*, indicating that AM has little intrinsic antimicrobial action without the inclusion of antibiotics during the manufacture process. To then investigate the role of the included antibiotics, we then decided to use bacterial strains that may show more resistance to the included antibiotic cocktail in disc diffusion assays. The results of these assays showed antibiotic processed VDAM has a variant ZOI on MRSA (10.74 mm) and MRSE (15.87 mm). Reduced activity was observed on *E*. *coli* (8.82 mm), *P*. *aeruginosa* (2.48 mm) and *E*. *faecalis* (1.96 mm) plates, but was still present and in a consistent manner. AM processed without antibiotic and/or raffinose were ineffective against MRSA, MRSE, *E*. *coli*, *P*. *aeruginosa* and *E*. *faecalis* strains with no ZOI, further indicating that washed AM untreated with antibiotic is not bactericidal.

Moreover, visual assessment of confluence of growth underlying the discs showed that bacteria did grow slightly underneath the control discs of plastic, PVDF and untreated AM, but not under VDAM and antibiotic-soaked filter paper. AM antibacterial action by adhesion was higher against gram-positive bacteria compared to gram-negative bacteria. This could be explained by the differences in defence mechanisms between both groups of bacteria^42^, and implies that fresh AM may have only a static antimicrobial action by adherence rather than a bactericidal effect, in agreement with Elian et al, 1991^40^. Placing fresh AM on PVDF membrane, to evaluate the biological antibacterial activity by anti-microbial peptide stripping, demonstrated that samples of AM without antibiotics have no such effects against any tested bacteria. These findings disagree with that of N. Kjaergaard *et al*., 2001, who showed that saline washed AM placed over a nitrocellulose paper was inhibitive to underlying bacterial growth of group B streptococcus, *Staphylococcus saprophyticus*, *P*. *aeruginosa* and *S*. *aureus*^43^.

The current study was limited to investigating whether the antibacterial action of the dried amnion was due to the intrinsic properties of amniotic membrane or due to the antibiotics added during processing. This study could have been further extended by investigating the individual activity of the antibiotics within the cocktail and by considering the effect of long-term storage on each of the individual antibiotics. The study could also be extended by investigating the anti-fungal properties of both amniotic membrane and the antibiotic/antimycotic cocktail added during processing.

AM processed under approved guidelines and as a licensed protocol results in better wound healing results and less post-transplant infections^15^. This effect is most likely due to antibiotic use, providing an antibiotic reservoir, substituting for the fresh AM’s lost antibacterial property and effective in prohibiting bacterial growth on wounds^44^. AM also functions as a physical barrier against infective pathogens^40,45^, with elastic, pliable properties allowing it to adhere completely to wounds^23^, preventing external infection recurrence.

The risk of major bioburden contamination of caesarean section collected AM is low. Nevertheless, the combined AM processing procedure used in this paper is a robust method to remove any bioburden, sub-clinical infection, and manufacturing-related contamination. Non-antibiotic processed AM may be bacteriostatic but has no bactericidal effect. The specific VDAM processing steps of washing in NaCl, spongy layer removal, raffinose incubation and drying, all contribute to significantly reduce the bacterial load, with drying and antibiotic treatment being considered a sterilisation step. The antibiotic reservoir of VDAM can eliminate natural and acquired contamination of gram-negative and gram-positive bacteria, such that VDAM is a reliably sterile product suitable for clinical application. Combined with vacuum packing, VDAM can be confidently and stably stored long-term without the risk of infection-related degradation.

## Methods

### Human amniotic membrane procurement and preparation

This study was carried out with the approval of the Nottingham Research Ethics Committee and the study (OY110101) complied with the tenets of the Declaration of Helsinki. Human placental samples were collected from consenting donors undergoing elective caesarean sections. Patients not complying with the inclusion criteria for tissue donation or having a history of antenatal problems were excluded. Written informed consent from the donor was obtained for sample use and the placenta membranes were prepared according to previously published methodology^46^.

### Identification of the natural flora of amniotic membrane natural flora

AM samples (separated from the chorion membrane) from 10 different donors were studied for the natural bacterial flora contamination. Unwashed AM samples were collected in 5 mL PBS (Gibco®, ThermoFisherScientific, UK) before sonication for 5 minutes. 200 µL of each sample was plated onto blood agar plates (Blood Agar Base No. 2 with sheep blood, Oxoid, UK) and incubated aerobically for 48 hours and anaerobically for 7 days at 37 °C, before colony forming units (CFU)/mL were counted. 100 µL volumes of sample were inoculated into Anaerobic Basal Broth (Oxoid), for 48 hours and 7 days at 37 °C in aerobic conditions to enhance bacterial proliferation. Bacterial growth was identified by turbidity and API® Staph Rapid 32 A microbial identification kit (BioMerieux, UK) was used to identify microorganisms.

### Vacuum-dried amniotic membrane (VDAM) processing

AM was separated from the chorion membrane by finger blunt dissection and triple washed in 250 mL 0.9% sodium chloride (NaCl) solution (Baxter, UK) for 15 minutes with rocking agitation to wash out blood. The spongy layer was removed by scalpel blade size 22 attached to a custom-made tool. The AM were soaked in 100 mM raffinose pentahydrate (Acros Organics, UK) solution in DPBS containing 5 mL of a broad-spectrum antibiotic/antifungal combination (Source Bioscience, UK, see Table 2) and incubated at 37 °C for 2 hours. To prepare for vacuum-drying, the AM was spread out epithelial side down over a sheet of parafilm and placed in a freeze dryer (Alpha 1–4 LSC, Advanced Freeze Dryer Christ® Osterode, Germany). Prior to sample drying the condenser was cooled to −45 °C to allow for vapour removal. Drying cycles consisted of a main dry phase of 30 minutes with a shelf temperature of 15 °C and vacuum pressure of 1.03 mbar followed by a final drying phase with a shelf temperature of 15 °C and vacuum pressure of 0.001 mbar for 15 minutes. After drying, AM was cut to the desired size and individually vacuum-packed in plastic pouches (Orved Multiple 315 Vacuum Sealer, Italy).

### Validation of VDAM decontamination process

#### Bacterial culture

A pure culture of a clinical isolate of *S*. *epidermidis* was transferred from a BA plate into 20 mL universal tubes containing Tryptone Soya Broth (TSB) (Casein Soya Bean Digest Medium, CM 0129, Oxoid) and incubated overnight at 37 °C. The next day absorbance at 490 nm was measured using a Jenway 6705 UV/Vis Spectrophotometer (Fisher Scientific, Leicestershire, UK) and the bacterial suspension adjusted to 10^8^ CFU/mL.

#### Bacterial loading of AM

Artificial loading of *S*. *epidermidis* was performed individually at three different stages of the VDAM process to assess the efficiency of the decontamination process at different steps (Fig. 1). To assess the entire process, the bacteria were loaded immediately after chorion removal and the samples taken and assessed for CFU at each stage. To assess the raffinose/antibiotic cocktail wash, the bacteria were loaded after spongy layer removal. To assess the drying stage bacteria were loaded after the incubation in raffinose (with/without antibiotic) and before vacuum drying. Sampling of unloaded and loaded tissues was performed throughout processing. Discs of 25 mm diameter were cut after collection of the AM. Bacteria inoculation was applied before each of the examined processing steps. To load the discs with *S*. *epidermidis*, a 2 mL microcentrifuge tube containing the AM sample in 1 mL PBS was inoculated with 100 µL of 10^8^ CFU/mL *S*. *epidermidis* and agitated by rolling for 1 hour at 4 °C.

#### CFU counting

Loaded and fresh AM samples in 1 mL PBS were twice diluted to give 10^−1^ and 10^−2^ dilutions of each sample, then 200 µL of each dilution was spread onto each of three BA plates. The plates were incubated for 48 hours aerobically (37 °C). The average of viable colonies was then counted and expressed as CFU/mL.

#### Minimum inhibitory concentration/minimum bactericidal concentration

Dry AM discs from 3 donors were prepared with and without antibiotics. Proteins were then extracted from the tissue by grinding with pestle and mortar in a liquid nitrogen bath. AM extracts were then tested against 10^6^ CFU/mL of gram-positive; *Staphylococcus aureus* (*S*. *aureus*), *S*. *epidermidis*, *Corynebacterium diphtheriae*, and gram-negative *Moraxella lacunata* bacterial strains. 50 µL/well of lysogeny broth (LB) medium (Sigma-Aldrich) was added to appropriate wells of a 96-well plate, followed by 50 µL of AM extract at concentrations of 100, 50, 25, 12.5, 6.25, 3.13, 1.56, 0.78, 0.39, 0.20, and 0.10 µg/mL loaded in triplicate across the plate. Culture medium only and medium with 100 mg/L gentamicin were used as negative and positive controls respectively. The bacterial strains were grown up in LB medium until optical density = 0.1 at 600 nm. 50 µL/well of the bacterial cell suspension added to each well of the plate. Plates were incubated overnight at 200 rpm on a rocker at 37 °C. Results were assessed visually with the minimum inhibitory concentration (MIC) equivalent to the last clear well, where bacterial growth is completely inhibited, and the minimum bacterial concentration (MBC) equivalent to the lowest concentration of test article required to kill 99.9% of the initial bacterial inoculum.

#### Disc diffusion assay

The following AM samples were prepared for disc diffusion assays; VDAM processed with raffinose and antibiotic (VDAM), VDAM processed in raffinose without antibiotic (R) and VDAM without raffinose or antibiotic (F). All samples were cut into discs of 10 mm diameter and kept in vials at 4 °C for a maximum of 4 days before the experiment was performed. Cultures of multidrug resistant *S*. *aureus* (MRSA), multidrug resistant *S*. *epidermidis* (MRSE), *Enterococcus faecalis*, *Escherichia coli*, and *Pseudomonas aeruginosa* bacteria were prepared from fresh pure cultures in TSB. The viable bacterial count of each was adjusted to 10^8^ CFU/mL using 0.5 McFarland standard. Each of the five bacterial cultures were seeded onto three Mueller-Hinton agar (MHA) (Oxoid) plates in 90 mm Petri dishes using a sterile cotton swab and subsequently the AM discs were aseptically placed on the even lawns of the bacterial strains. An antibiotic-soaked disc of Whatman no.3 filter paper (AB) was performed as a positive control and a disc of transparent laminating plastic (P) was used as a negative control. Three additional discs were also assessed, using hydrophobic highly stripping polyvinylidene fluoride (PVDF) transfer membrane discs, pore size 0.45 µm (Hybond™-P, Amersham Biosciences, UK), one of which was used alone as a control (PV). The PVDF was then used to separate the MHA surface and VDAM-R (R/PV) and VDAM-F (F/PV) to assess biological antibacterial activity, rather than physical blocking. MHA plates were incubated at 35 °C for 18–24 hours and zone of inhibition (ZOI) were measured in mm using a digital calliper (Mitutoyo Absolute Digimatic Caliper, UK). To confirm whether there was any bacterial growth beneath the samples, sterile disposable loops were used to touch areas under the discs of the same bacterial plates and streak on another Tryptone Soya Agar (TSA, Casein soya bean digest agar, CM0131, Oxoid) plate before overnight incubation at 35 °C.

To specifically investigate time line of antibiotic release from VDAM samples, ZOI were measured at 0, 6, 12 and 24 hours.

#### Sterility test of VDAM held in long-term storage

Triplicate samples of 25 mm diameter (average storage time 757 days, 7 donors each with 3 samples) and 10 mm diameter (average storage time 862 days, 10 donors each with 3 samples) VDAM discs were tested for sterility. Discs were either aseptically immersed in falcon tubes containing 40 mL TSB for aerobic bacteria, facultative bacteria and fungus, or placed in thioglycolate broth medium U.S.P. (CM0173, Oxoid) for anaerobic and facultative bacteria. TSB tubes were incubated at 35 °C with shaking (80 rpm), for 21 days along with negative and positive control tubes. Thioglycolate medium tubes were incubated for 21 days at 35 °C without shaking. Turbidity was used as an indicator of non-sterility and was checked daily. Streaking of 10 µL TSB from each tube was performed weekly on TSA plates, which were subsequently incubated for 5 days at 37 °C and observed for bacterial growth.

### Statistical analysis

Statistical analysis was performed using GraphPad Prism version 7.01.

## Data Availability

The datasets generated during and/or analysed during the current study are available from the corresponding author on reasonable request.
